# Resolution of late‐onset gastro‐colic fistula after laparoscopic sleeve gastrectomy by conservative management: a case report

**DOI:** 10.1002/ccr3.1551

**Published:** 2018-05-31

**Authors:** Julien‐Sami El Sayegh, Gregory Nicolas, Kabalan Yammine, Claude Tayar

**Affiliations:** ^1^ Lebanese American University Medical Center – Rizk Hospital Beirut Lebanon; ^2^ Clemenceau Medical Center Beirut Lebanon

**Keywords:** bariatric surgery, gastro‐colic fistula, gastroenterology, general surgery, nutrition, sleeve gastrectomy

## Abstract

Nutritional support and Antibiotics treatment can be used as conservative treatment for the resolution of gastro‐colic fistula after sleeve gastrectomy in stable patients specially to prevent cumbersome redo surgeries that have higher risks of complications particularly in patients with minimal financial means.

## Introduction

Gastro‐colic fistula is a known complication after sleeve gastrectomy. Late‐onset GC fistula (> 12 weeks) is very rare and not frequently reported. Usually managed by surgical means with a roux‐en‐Y esophagojejunostomy, or endoscopic interventions. This is the first case to be reported that was managed conservatively.

Obesity is a disease that has become an epidemic all around the world. With numbers reaching more than 600 million overweight adults of whom 400 million are clinically obese, this disease needs to be diagnosed and treated accordingly 1. Bariatric surgery has shown tremendous advantage with weight loss and obesity‐related morbidity reduction over conservative treatment (diet and exercise) that is considered first line treatment in the majority of obesity cases.

Laparoscopic sleeve gastrectomy (LSG) originally described by Marceau et al. 2 in 1993 as a restrictive part of duodenal switch malabsorptive operation has gained tremendous reputation in the last decade as an efficient stand alone restrictive procedure for weight loss in patients with obesity or morbid obesity. It involves resection of 70–75% of the gastric volume starting about 4–5 cm from the pylorus and proceeding toward the gastro‐esophageal junction, thereby creating a long gastric tube, accelerating gastric emptying, and removing the body and fundus of the stomach containing hunger hormone‐secreting cells (ghrelin) 1. Two of the most frequent complications of this procedure are staple line bleeding and staple line gastric leak. The latter is one of the most dreaded complications by most surgeons occurring usually at the upper part of the staple line near the gastro‐esophageal (GE) junction 3. If not diagnosed and treated promptly, a gastric leak may lead to severe abdominal sepsis and may progress to multi‐organ failure and death, or they may progress to chronic gastric fistulas 3. Leaks are classified as early (1–3 days after surgery), intermediate (4–7 days after surgery), and late (8 days ≥ after surgery) 4. Among the reported gastric fistulas in the literature, we mention gastro‐cutaneous, gastro‐bronchial, gastro‐pleural (GP), and gastro‐colic fistulas (GC). To our knowledge, no case of late‐onset GC fistula following primary LSG managed conservatively has ever been reported in the literature, some articles report early GC fistula and others late GC fistula all managed surgically. Therefore, we present a case of late‐onset GC following LSG that was managed by medical therapy until its resolution.

## Case Presentation and Management

**Figure 1:**
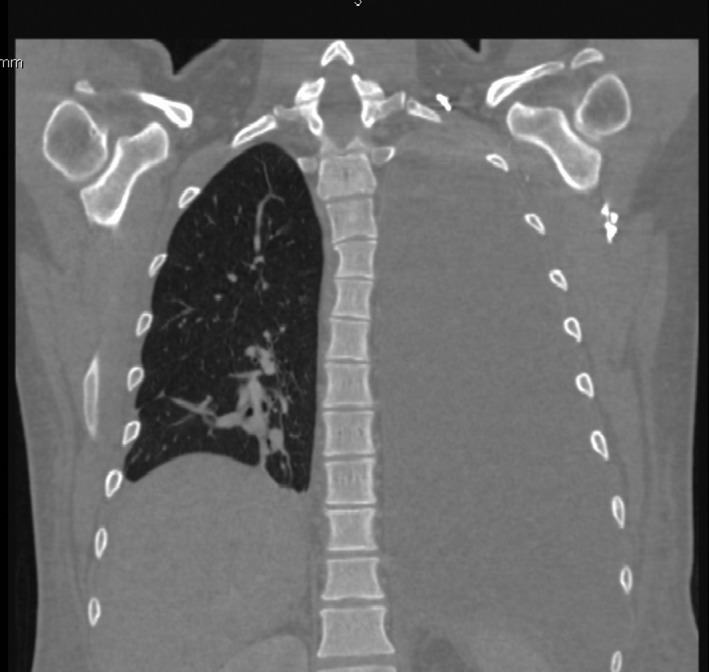
Left reactional pleural effusion day of admission.

**Figure 2:**
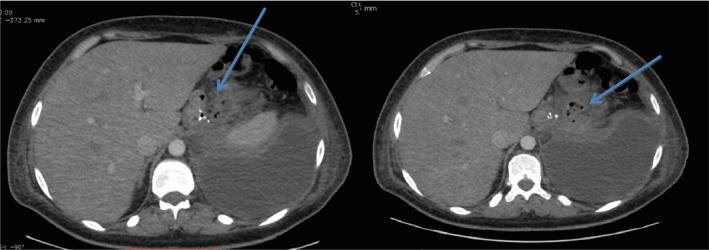
CT scan abdomen showing left upper quadrant staple line leak corresponding to the gastro‐colic fistula (arrows point to fistulous tract), note the left pleural effusion.

**Figure 3:**
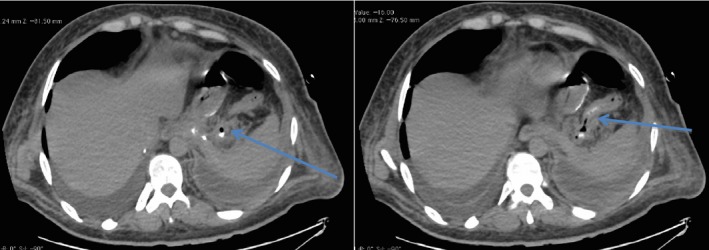
CT abdomen with oral contrast showing leak of oral contrast from the gastric pouch into the distal transverse colon (arrows point to the fistula), note resolution of left pleural effusion.

**Figure 4:**
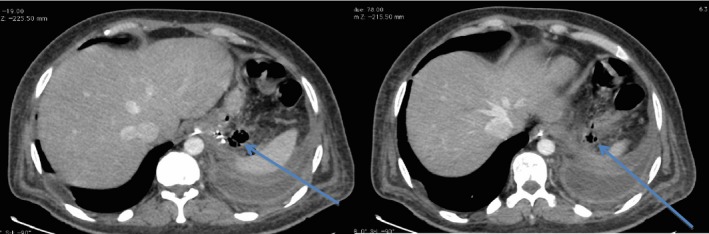
CT scan 10 days after admission with persistence of gastro‐colic fistula with evident air bubbles at the site of fistula (indicated by arrows).

**Figure 5:**
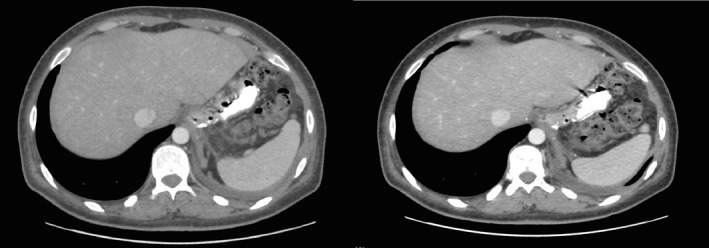
CT scan abdomen with PO contrast showing resolution of the gastro‐colic fistula showing continuous staple line with no extravasation of contrast.

Our case was a 28‐year‐old male heavy smoker with morbid obesity (initial weight = 150 kg, height = 180 cm BMI = 46 kg/m^2^) that was operated with a LSG at another institution with a stable postoperative course. He reported chronic dysphagia and vomiting despite normal postop imaging (upper GI series and CT scan). Patient has lost the foreseen weight at 6 months (50 kg, BMI = 30.86 kg/m^2^, %EWL = 72.5%), but he presented to our institution 8 months later with a left pleuritic chest pain, nonradiating in nature associated with left shoulder pain, dyspnea, fever, chills, and decrease food intake (he stopped eating well 2 weeks prior to presentation because of described odynophagia and took multiple doses of IV NSAIDS). Upon admission, patient was hypotensive with a deteriorating general status (anxious, nadir GCS was 11, hypotensive, tachycardic) and a bad respiratory status (dyspnea, tachypnea, crackles on physical exam specially in the left side, with decrease left sided air entry). A chest x‐ray showed a left massive pleural effusion with left lower lobe consolidation (effusion + pneumonia) (Fig. 1). In an effort to explain the cause of the effusion, a CT scan chest, abdomen and pelvis with IV and oral contrast, showed a proximal stomach (gastro‐esophageal junction) staple line fistula that was draining to what was first described as a left upper quadrant abscess later found to be a GC fistula causing a left pleural effusion (reactional effusion) (Figs. 2 and 3). He had a chest tube placed immediately that drained nonpurulent sero‐sanguinous liquid of which one set of culture was taken. He was then started on empiric antibiotics, ceftriaxone, and levofloxacin, after cultures were taken and the patient was kept NPO. Four days later, chest tube was removed, and conservative management was continued. Total parenteral nutrition was started because the patient was malnourished, with low serum albumin, low iron, and evident muscular wasting (although he maintained a BMI of 26). Results of new cultures were out and showed no growth. Patient's antibiotics were switched to piperacillin/tazobactam and fluconazole. He was scheduled for a salvage surgery with a laparoscopic esophagogastrectomy with a Roux‐en‐Y esophagojejunal reconstruction when his nutritional status was adequate enough for the procedure, and after resolution of his infection. Ten days after, a repeat CT scan was done that showed a smaller residual left pleural effusion plus enhancement of the visceral and peritoneal pleura, and persistence of the gastro‐colic fistula (Fig. 4). GP fistula was excluded and GC fistula was confirmed. Patient was discharged home with a naso‐jejunal tube (placed 1 day before discharge) for enteral feeding with high protein shakes (1500 mL Fresubin HP and 10 scoops of Protiphar/day to compensate for his daily caloric needs of 2400 kcal per day) and was kept on PO antibiotics (co‐amoxiclav). A total of 14 days of IV antibiotics and another 2 weeks of PO antibiotics were taken. Upon many outpatients’ consultations, the patient family was concerned with the possible risks and benefits of the reoperation that was thoroughly explained to them and to the patient with multiple counseling sessions. They opted to continue conservative treatment (antibiotics + naso‐jejunal enteral nutrition) in an attempt for the fistula to resolve without surgery because they feared the possible outcomes. The patient was carefully monitored over the course of 8 weeks with serial CT scans and outpatient visits, and finally on the last CT scan, the GC fistula had completely resolved (Fig. 5), with no evidence of pleural effusion and the patient was started on a progressive oral diet after removal of the Naso‐Jejunal tube. On future follow‐ups, the patient seemed to do well, with adequate nutrition and good quality of life.

## Discussion

Historically, GC fistulas were described as being usually the effect of a gastric or a colonic malignancy and other benign conditions such as peptic ulceration, Crohn's disease, chronic pancreatitis, and diverticular disease 5_._ Coronary artery bypass procedure involving the right gastro‐epiploic artery has been reported as a cause of GC fistula 5. Two cases of GC fistula after bariatric surgery have been reported, one was after a gastric band slippage into the colon, and the other after a resleeve gastrectomy that was treated through endoscopic stenting + laparoscopic repair of the fistula. 5 No GC fistula following primary laparoscopic sleeve gastrectomy has been reported until in 2015, Nguyen et al. 6 presented three case series with complex fistulas treated with surgical intervention (en bloc resection of fistula with proximal gastrectomy and Roux‐en‐Y esophagojejunostomy), with one case being a GC fistula occurring in a 21‐year‐old male 6 months after LSG. Recently in 2017, Khuri et al. 7 reported a successful endoscopic management of a GC fistula using over the scope clip closure of the fistula opening on the stomach side and a heme clip applied at the opening of the fistula at the colonic side with distal gastric stenosis balloon dilatation. In the early stages of any gastric leak after bariatric procedure, the initial management is nonoperative, if the patient is hemodynamically stable. When signs of instability and diffuse peritonitis appear, they require immediate intervention. The unique physiologic features of LSG (increased intraluminal pressure, intact pylorus distally, narrowing at the level of the incisura) sometimes influence the persistence of small postoperative leaks and their progression to chronic fistulas (> 12 weeks) 6_._ Late‐onset fistulas seldom close on their own, and treatment of choice is surgical intervention after optimization of nutritional status. The peculiar feature of this case report is the late‐onset of the GC fistula (8 months after the initial operation) with no prior symptomatology, and no immediate postoperative signs of gastric leak. Also what is of interest is the success of conservative management in treating what was at one time a serious symptomatic GC fistula with deleterious consequences. This is the first late‐onset GC fistula following LSG treated conservatively to be reported in the literature.

In a nutshell, a few cases of chronic gastric fistula may heal by conservative management with drainage, nutritional optimization, and antibiotics treatment, thereby preventing a high morbidity (50%) and mortality (2–10%) surgical procedures 6.

## Conclusion

Late‐onset gastro‐colic fistula (> 12 weeks) is a rarely reported complications and usual management reported has been through surgical en bloc resection of the fistula with a Roux‐en‐Y esophagojejunostomy. Recent reports of endoscopic managements have been also successful. This is the first conservatively managed late‐onset GC fistula following LSG to be reported that has been successful. Conservative management with nutritional support administered beyond the fistula site and antibiotics treatment has proven to be beneficial in treating and resolving a once symptomatic gastro‐colic fistula in hemodynamically stable patients. This opens up a new perspective in treatment of such a complications especially in patients that are resistant to surgical approach and with minimal financial means to afford a redo surgery (that carries high complication risks) or a financially burdening endoscopic procedure particularly in the Lebanon and middle east region, where not all individuals are medically insured, and where such complex surgical procedures could be very costly for the average citizen.

## Conflict of Interest

None declared.

## Authorship

J‐SES: General surgery resident, Lebanese American University Medical Center – Rizk hospital, Beirut, Lebanon: first corresponding author, gathered all the information, documents and illustrations regarding the case as well as was on board in the management of the patient during his hospitalization, did the literature review, wrote the manuscript. GN: General surgery resident, Lebanese American University Medical Center – Rizk hospital, Beirut, Lebanon: helped me in the literature review, contributed to the writing of the introduction and parts of the case presentation. KY: Radiology attending, Clemenceau Medical Center, Beirut, Lebanon: contributed in the analysis of the imaging and selection of the cuts that were used in the manuscript and guided me in the literature review. CT: Professor of General surgery and head of department, Clemenceau Medical Center, Beirut, Lebanon: The main attending physician on the case. He prompted me to think about the topic, as he is an expert in the bariatric surgery field especially postoperative complications and their surgical managements. He enlightened me with information from his personal experience and he also provided me with reading material to prepare for the topic. He also reviewed my manuscript before submission to the journal.
